# Influence of Annealing and Aging Parameters on the Microstructure and Properties of 1200 MPa Grade Cold-Rolled Dual-Phase Steel

**DOI:** 10.3390/ma17194933

**Published:** 2024-10-09

**Authors:** Xiaoyue Ma, Xiaohong Chu, Yuebiao Yang, Hongzhou Lu, Wenjun Wang, Zhengzhi Zhao

**Affiliations:** 1 Collaborative Innovation Center of Steel Technology, University of Science and Technology Beijing, Beijing 100083, China; m202221344@xs.ustb.edu.cn (X.M.); chuxh1118@163.com (X.C.); 2 Beijing Engineering Technology Research Center of Special Steel for Traffic and Energy, University of Science and Technology Beijing, Beijing 100083, China; 3 Guangxi Liuzhou Iron and Steel Group Co., Ltd., Liuzhou 545002, China; yangyuebiao@gmail.com; 4 CITIC Metal Co., Ltd., Beijing 100004, China; luhz@citic.com (H.L.); wangwj3@citic.com (W.W.)

**Keywords:** cold-rolled dual-phase steels with high formability, microalloying, annealing temperature, over-aging temperature, fracture behavior

## Abstract

With the rapid development of the automotive industry, the requirements for bodywork materials are not only focused on high strength but also on improved forming properties. To develop a new generation of automotive steels with higher strength–plasticity matching, a high elongation 1200 MPa grade V-Nb microalloyed cold-rolled reinforced formable dual-phase steel was developed in this experiment through rational compositional design and precise process machining. The properties of the test steel are improved by varying the over-aging temperature as well as the annealing temperature to achieve a good strength–plasticity balance. The results show that as the aging temperature increases, the tensile strength and yield strength of the test steel decrease, while the elongation continues to increase. At an aging temperature of 310 °C, the steel exhibits not only high strength but also better ductility. As the annealing temperature increases, the tensile strength and yield strength of the test steel initially increase and then decrease, while the elongation continues to increase. When the heat treatment process involves an annealing temperature of 860 °C and an over-aging temperature of 310 °C, the test steel achieves the best strength–plasticity balance.

## 1. Introduction

Guided by the dual-carbon target, the automotive industry is moving towards weight reduction, environmental protection, and energy saving. Lightweighting of vehicles is an effective method to improve safety, energy efficiency, and emission reduction. For every 10 percent reduction in the weight of a vehicle, fuel consumption per unit distance is reduced by 8 percent, with a corresponding 4 percent reduction in emissions. The use of high-strength steel plates not only reduces body weight and fuel consumption but also ensures safety [1]. Therefore, the development of a new generation of thinner, more moldable, and stronger materials has become the goal of the automotive industry. Dual-phase steel is obtained from low-carbon or low-carbon low-alloy steel by annealing in the two-phase zone or through controlled rolling and cooling. Its main microstructure consists of ferrite and martensite, which provides the advantages of a good strength–ductility match, a low yield-to-strength ratio, and continuous yielding. This has made it a key material in modern transportation steel [2,3,4,5]. Dual-phase steels with high formability (DH) are made by adding retained austenite to conventional dual-phase steels to produce a TRIP effect during deformation [6,7]. This improves the strength of automotive plates while also providing significant molding capability. The main process characteristics of continuous annealing for the production of DH steels are the following: rapid heating to the critical zone, critical zone holding, two-stage cooling, and over-aging treatment [8]. The two-stage cooling process includes a slow-cooling section and a fast-cooling section. The slow-cooling section facilitates the further cementite, improving the purity of ferrite and the hardenability of austenite. The fast-cooling section ensures that the undercooled austenite is fully transformed into martensite. The role of the over-aging process, on the other hand, is to temper the hardened martensite in the steel, reducing its hardness and improving the overall mechanical properties.

The properties of DH steels mainly depend on the distribution of ferrite and martensite microstructures, grain size, and proportional morphology [9]. The main parameters affecting the microstructure of DH steels are the annealing temperature in the two-phase zone, the holding time, and the over-aging temperature [5,10,11,12]. Researchers have continuously increased the alloying element content in DH steels to enhance their strength; however, the elongation has struggled to meet the corresponding requirements [13,14]. Currently, the addition of the element Nb to steel is more widely used, while research on the element V is relatively less.

Nb delays the transformation of ferrite and pearlite during the slow-cooling process and refines the original austenite grains in dual-phase steels [5,15]. Relevant studies have shown [15,16,17,18,19] that the strengthening effect of the element V on steel is stronger than that of the element Nb. The addition of V can hinder the growth of austenite grains, expand the temperature range of the two-phase zone, and the precipitated phase of V can play a role in precipitation strengthening. Additionally, the unprecipitated V in austenite can also improve hardenability [11,20,21]. Therefore, to further improve the elongation of the test steel and enhance its strength–plasticity match in order to develop a lighter and safer steel for automotive use, the test steel was designed with a rational composition. By adding V, Nb, and other alloying elements to refine the microstructure, and simultaneously considering actual production conditions, different over-aging and annealing temperatures were formulated to produce dual-phase steel with high strength and plasticity matching.

## 2. Materials and Methods

The chemical composition of DH steel is usually based on a low-carbon and low-alloy system of C, Si, and Mn [3], with an appropriate amount of alloying elements added to improve the performance stability and enhance the strength and plasticity matching. The chemical composition (mass fraction, %) of the test steel for this experiment is shown in Table 1.

The test steel was smelted, cast, and forged to obtain a forging billet with dimensions of 30 mm × 80 mm × 100 mm. The hot rolling simulation test first places the forged billet in a high-temperature muffle furnace at 1200 °C for 1 h of soaking, with an initial rolling temperature of 1150 °C and a final rolling temperature of 890 °C. After five rounds of hot rolling, it is rolled to a thickness of about 3.5 mm. After the hot rolling is completed, the steel plates are immediately water-cooled to 700 °C and then placed in a muffle furnace at 700 °C to simulate coiling. After soaking for 1 h, they are cooled to room temperature in the furnace. The hot-rolled steel plate is acid washed to remove oxide scale, and then cold-rolled to about 1.6 mm.

A cylindrical thermal expansion specimen with dimensions of 4 mm in diameter and 10 mm in length was cut from the forging blank using a wire-cutting machine (WEDM DK7732, Ningbo Jiangbei CNC Machinery Co., Ningbo, China). The critical phase transformation point and continuous cooling transformation curve of the test steel were measured using the DIL805A dilatometer (Thermoanalyse GmbH, Hüllhorst, Germany) to provide a theoretical reference for the formulation of subsequent experimental plans. Experimental measurements show that the austenitization temperature *A_C_*_1_ during heating is 726 °C, the austenite transformation end temperature *A_C_*_3_ is 944 °C, the martensite transformation start temperature *M_s_* is 355 °C, and the martensite transformation end temperature *M_f_* is 224 °C.

**Figure 1:**
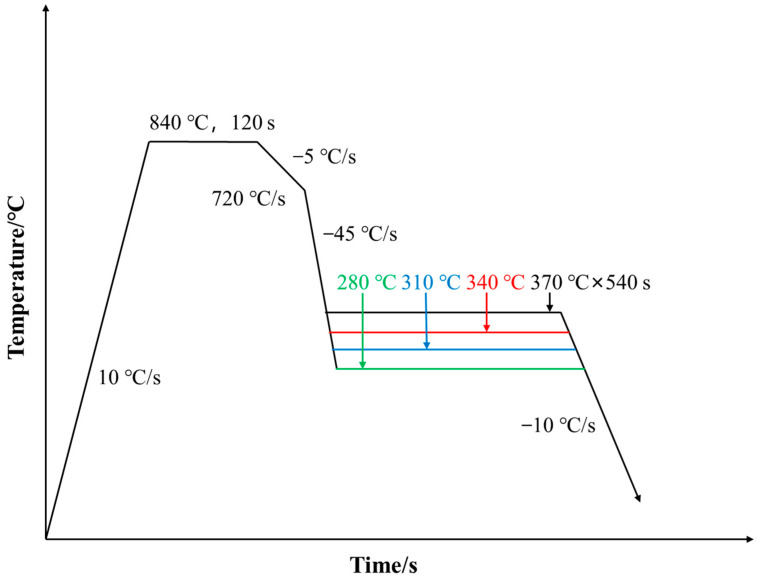
Continuous annealing process route.

Over-aging treatment in the actual production line is of significant importance for improving the material’s ductility and other comprehensive mechanical properties. This paper, according to the actual production situation, selected over-aging temperatures of 280 °C, 310 °C, 340 °C, and 370 °C for the test steel, with an annealing temperature of 840 °C. The process routes are shown in Figure 1.

Annealed specimens measuring 190 × 50 mm were cut from cold-rolled plates and subjected to simulated continuous annealing using the CCT-AY-II (Ulvac-Riko INC, Tokyo, Japan) continuous annealing simulation tester. Combined with the critical phase change point temperature determined by the dilatometry method and actual industrial production conditions, the two-phase zone heating temperature and over-aging temperature were set accordingly.

A_50_ tensile specimens with a gauge length of 50 mm were cut from the annealed plate in the rolling direction, and the mechanical properties of the specimens were tested using a tensile testing machine (MTS Systems Corporation, Eden Prairie, MN, USA) at a tensile rate of 2 mm/min. The metallurgical specimens were cut from the annealed plate and mechanically polished after being sanded with sandpaper for several passes up to 2000 mesh. Subsequently, the specimens were etched with a 4% nitric acid–alcohol solution and the microstructure was observed using a ZEISS Gemini 500 field emission scanning electron microscope (SEM, Gemini SEM 500, Oberkochen, Germany). Backscattered electron acquisition specimens were electrolytically polished with 10% nitric alcohol and then examined under a scanning electron microscope equipped with an EBSD probe (Symmetry S2, Oxford Instruments, Abingdon, UK) at 20 kV and a step size of 0.07 μm. The data were processed using AZtecCrystal software v2.1.2 to observe the grain size, orientation, and distribution of retained austenite. Transmission electron microscopy specimens were prepared by carbon extraction replication, and were subjected to grinding, polishing, etching, carbon coating, and carbon film separation processes. The specimens were examined using a JEM-2100 transmission electron microscope (Tokyo, Japan) to observe the second-phase precipitates and to measure their size using Image-Pro Plus v6.0 software. The volume fraction of retained austenite was determined by XRD analysis using a Bruker D8 Advance diffractometer (Karlsruhe, Germany) with Cu Kα radiation. The samples were scanned over a 2θ range of 40° to 95°, and the data were analyzed using Jade v6 software. The diffraction pattern was obtained using an X-ray diffractometer (Bruker D8-Advance diffractometer, Karlsruhe, Germany), and then X-ray analysis software Jade v6 was used to identify peaks. The {200}, {220}, and {311} diffraction lines of austenite, as well as the {211} and {200} diffraction lines of ferrite, were selected and calculated using the following formula [22]:

Vγ = 1.4Iγ/(Iα + 1.4Iγ)(1)

where Vγ is the volume fraction of retained austenite; Iγ is the average integrated intensity of the diffraction peaks of austenite {200}, {220}, and {311} crystal planes; Iα is the average integrated intensity of the diffraction peaks of ferrite {211} and {200} crystal planes. The carbon content of the retained austenite is calculated as follows [23]:

W(c)γ = (aγ − 3.547)/0.046(2)

where W(c)γ is the mass fraction of carbon in retained austenite (%); aγ is the lattice constant of retained austenite.

## 3. Results and Discussion

### 3.1. Effect of Over-Aging Temperature on Microstructure

**Figure 2:**
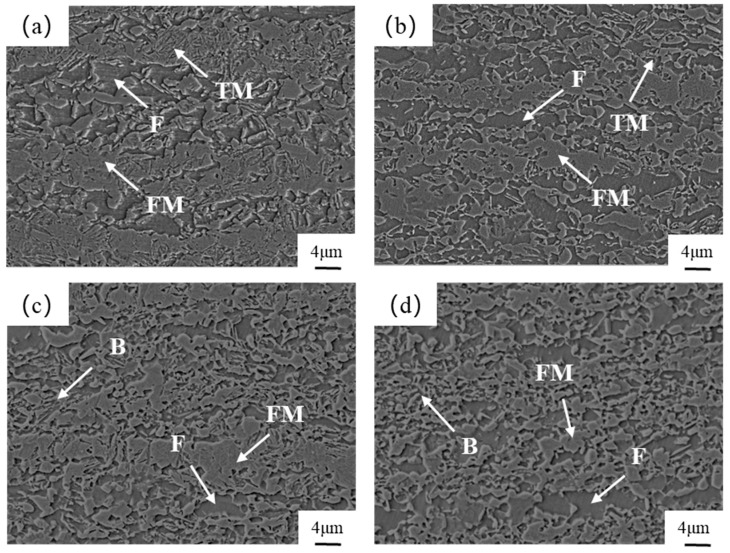
Microstructure of the test steel at different over-aging temperatures: (**a**) 280 °C; (**b**) 310 °C; (**c**) 340 °C; (**d**) 370 °C. (F) ferrite; (FM) quenched martensite; (TM) tempered martensite; (B) bainite.

Figure 2 shows the microstructure of the test steel at different over-aging temperatures, which is mainly composed of ferrite (F) and quenched martensite (FM) [24]. When the over-aging temperature is 280 °C, the ferrite and martensite microstructure is distributed in bands, and part of the martensite has been tempered [25]. When the over-aging temperature is raised to 310 °C, the amount of martensite transformed from austenite during the rapid-cooling stage decreases, resulting in a decrease in the amount of tempered martensite (TM) formed during the over-aging stage. In the final cooling stage to room temperature, the untransformed austenite will either transform into martensite or remain as retained austenite [26,27]. When the over-aging temperature is increased to 340 °C, the amount of tempered martensite decreases but the degree of tempering increases, and a certain amount of bainite (B) is produced [28,29]. Continuing to increase the over-aging temperature to 370 °C results in more bainite transformation and finer grains. This is due to the rapid cooling, which forms martensite as a carbon (C)-supersaturated solid solution. During the aging process, the C element diffuses to the austenite or precipitates, resulting in the decomposition and reduction in size of the martensite. At the same time, the surrounding austenite experiences carbon enrichment, and the increase in the number of nucleation sites of the nascent structure during the isothermal process leads to an increase in the homogeneous refinement of the final microstructure [30,31].

**Figure 3:**
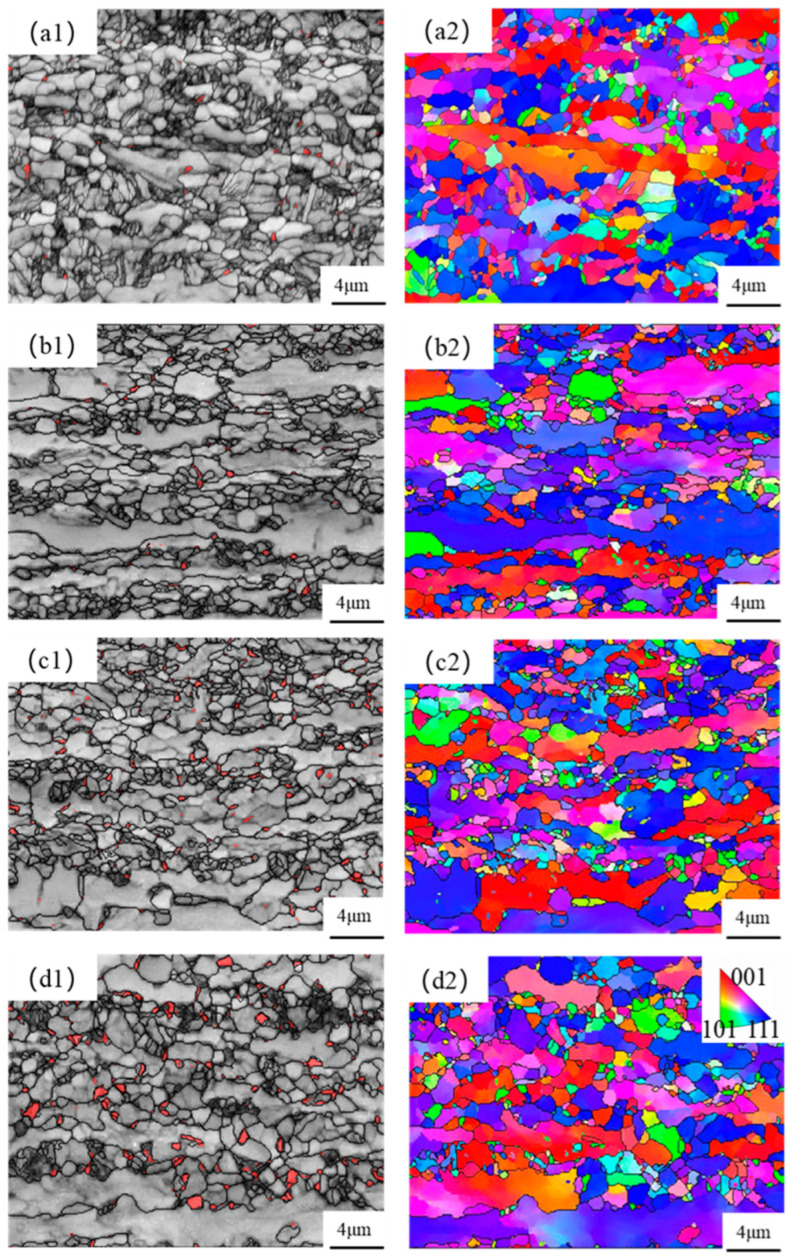
EBSD test results of test steels at different over-aging temperatures:(**a1**,**a2**) 280 °C; (**b1**,**b2**) 310 °C; (**c1**,**c2**) 340 °C; (**d1**,**d2**) 370 °C; (−1) BC and retained austenite phase diagrams; (−2) IPF.

**Figure 4:**
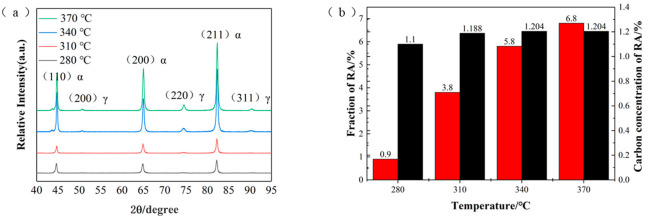
XRD results of the test steel at different annealing temperatures. (**a**) XRD patterns; (**b**) volume fraction of retained austenite and carbon content.

The EBSD characterization of the test steel at different over-aging temperatures is shown in Figure 3. In the band contrast (BC) and retained austenite phase, the red areas represent the retained austenite phase. It can be observed that the amount of retained austenite increases with the rise in over-aging temperature and is mainly distributed at the grain boundaries. This is because, with the increase in over-aging temperature, the amount of martensite formed during the rapid-cooling stage decreases, leading to more retained austenite. Inverse pole figure (IPF) maps show the crystal orientation of each grain in the microstructure. It can be seen from the IPF diagram that banded ferrite still exists at the annealing temperature of 840 °C, but the grain orientation of the microstructure with high-temperature over-aging is more uniform. To further investigate the retained austenite content of the test steels at each over-aging temperature, the specimens were characterized by XRD [32], as shown in Figure 4. From the figure, it can be seen that the peak value at high-temperature over-aging is significantly higher than that at low-temperature over-aging. The retained austenite content of the test steel is calculated to increase from 0.9% at 280 °C to 6.8% at 370 °C. Additionally, the carbon content in the retained austenite increases, which contributes to the enhancement of elongation.

### 3.2. Effect of Over-Aging Temperature on Properties

**Figure 5:**
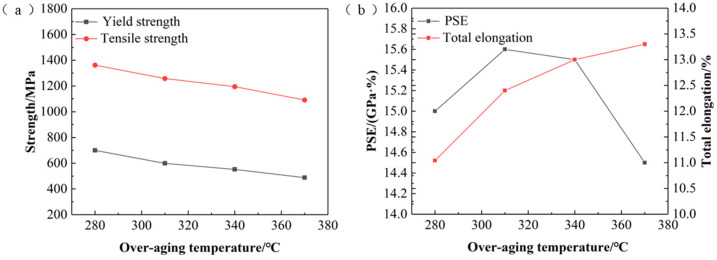
Mechanical properties of the test steel under different over-aging temperatures. (**a**) The relationship between over-aging temperature and strength; (**b**) the relationship between over-aging temperature and the product of strength and elongation, and the relationship between over-aging temperature and elongation.

The comparison of the mechanical properties of the test steels at different over-aging temperatures is shown in Figure 5. It can be seen that as the over-aging temperature increases, the tensile and yield strengths of the test steels decrease, the elongation increases, and the yield–strength ratio decreases. Excellent properties were obtained at an aging temperature of 310 °C, where the elongation was 12.4% and the strength–ductility product (PSE) reached 15.6 GPa%. The larger yield stresses of the specimens at lower over-aging temperatures are mainly attributed to the fine martensitic laths as well as the fine grain-strengthening effect of the V-Nb microalloying elements [33]. During the holding stage at lower over-aging temperatures, the degree of martensite tempering increases with increasing over-aging temperature, resulting in tissue softening [34]. During the holding stage at higher over-aging temperatures, the bainite transformation phase region is entered, and the bainite hinders dislocations less than the martensitic microstructure, thus reducing the yield strength. The increase in over-aging temperature allows the alloying elements to enter the austenite [35], which ensures the stability of the austenite. Due to the higher over-aging temperature, the quenched martensitic transformation decreases, and the increase in retained austenite content raises the elongation of the test steels while decreasing the yield and tensile strengths.

### 3.3. Effect of Annealing Temperature on Microstructure

**Figure 6:**
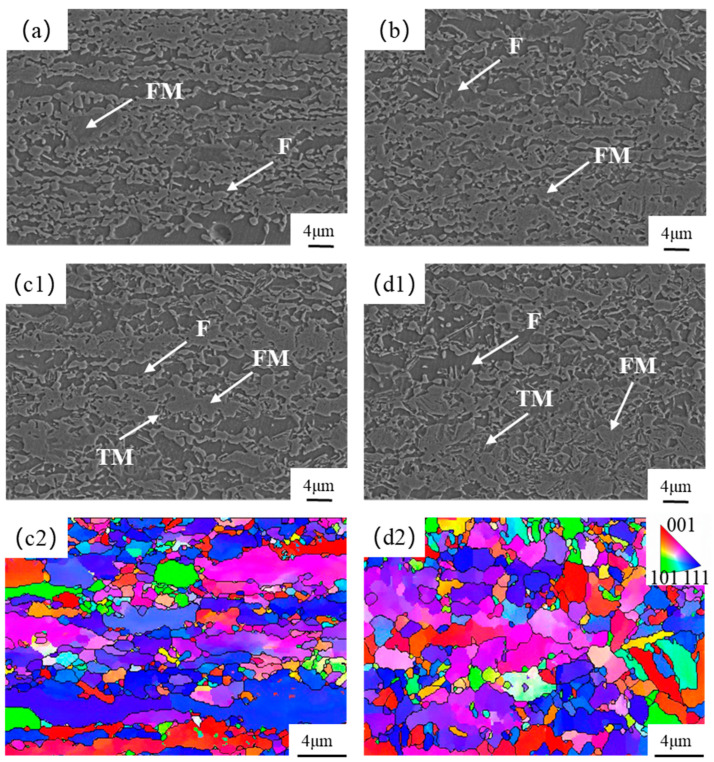
Microstructure and EBSD images of the test steels at different annealing temperatures: (**a**) 800 °C; (**b**) 820 °C; (**c1**,**c2**) 840 °C; (**d1**,**d2**) 860 °C. (F) ferrite; (FM) quenched martensite; (TM) tempered martensite.

In the previous section, it was mentioned that the test steel exhibited better properties at an over-aging temperature of 310 °C. Therefore, an over-aging temperature of 310 °C and annealing temperatures of 800 °C, 820 °C, 840 °C, and 860 °C were selected to anneal the test steel again. The annealing temperatures are based on the two-phase zone temperatures derived from the thermal expansion test performed earlier. To investigate which annealing temperatures result in better strength–plasticity matching for the test steel, Figure 6 shows the microstructure and the EBSD images of the test steel. At annealing temperatures lower than 820 °C, the degree of ferrite recrystallization is low, and the band microstructure formed by cold rolling is retained [36]. When annealing temperatures exceed 820 °C, the increasing degree of austenitization leads to a decrease in the average content of alloying elements in the austenite [37,38]. This results in decreased stability and the formation of more quenched martensite during the subsequent fast-cooling stage, which then transforms into tempered martensite during the over-aging stage [39]. It can be observed that at 860 °C, there is more tempered martensite compared to 840 °C, which can lead to a decrease in strength, as shown in Figure 6(c1,d1). From Figure 6(c2,d2), it can be seen that the grain orientation is more uniform at higher annealing temperatures.

**Figure 7:**
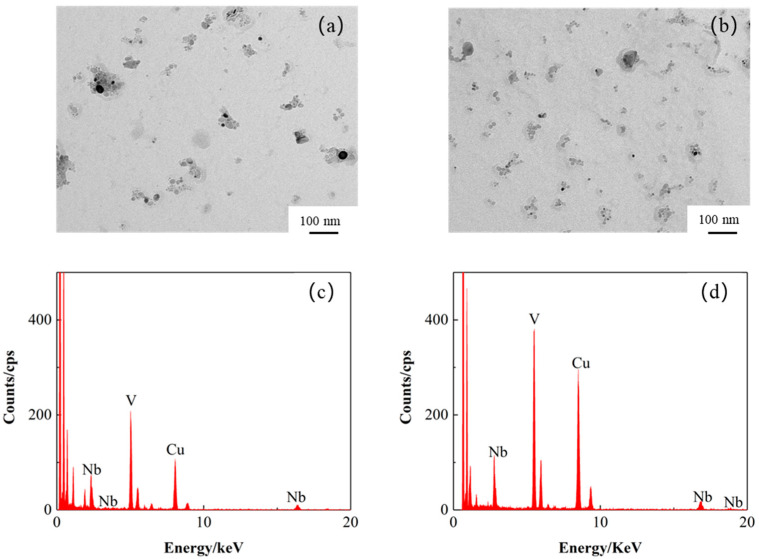
The morphology and energy spectra of the precipitates at 800 and 820 °C: (**a**,**c**) 800 °C; (**b**,**d**) 820 °C.

Transmission electron microscope (TEM) samples were obtained at 800 °C and 820 °C using the carbon extraction replication technique, and the precipitation of microalloying elements was investigated by TEM. The morphology of the precipitates is shown in Figure 7a,b, from which it can be seen that the sizes of the precipitates at both over-aging temperatures are in the nanometer scale, and all of them are V-Nb composite precipitates. The presence of the Cu elemental peak is due to the use of a Cu mesh as a carrier during the TEM test. Most of the precipitates were found to be less than 10 nm in size and diffusely distributed, as determined statistically using ImageJ. The number of precipitates at 820 °C is significantly higher than at 800 °C, and the size of the precipitates is more uniform and fine [40]. This increased number and uniformity of precipitates is one of the reasons for the increased strength of specimens annealed at 820 °C.

### 3.4. Effect of Annealing Temperature on Properties

**Figure 8:**
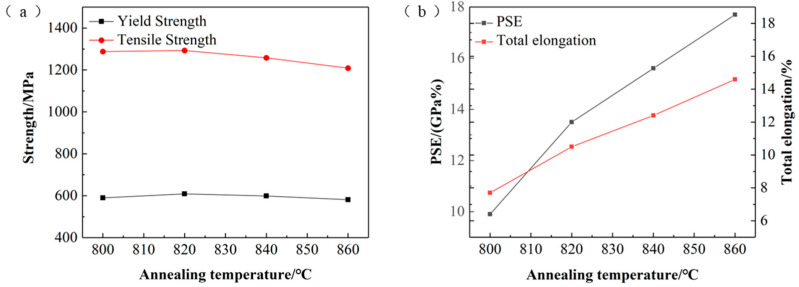
Mechanical properties of the test steel under different annealing temperatures. (**a**) The relationship between annealing temperature and strength; (**b**) the relationship between annealing temperature and the product of strength and elongation, and the relationship between annealing temperature and elongation.

The different heating temperatures in the two-phase zone mainly affect the degree of austenitization, which in turn affects the final proportion and distribution of ferrite and martensite, ultimately leading to different mechanical properties. Figure 8 shows the trend in the mechanical properties of the test steel under different annealing temperatures. With the increase in annealing temperature, the tensile strength and yield strength of the test steel initially increase and then decrease, while the elongation continues to increase. The highest elongation is exhibited at 860 °C, with a strength above 1200 MPa. Comprehensive microstructure and mechanical properties analysis shows that when heated at 800–820 °C, the degree of austenitization increases with the heating temperature. This results in an increased number of martensite formed during quenching, leading to higher specimen strength. Additionally, the addition of V and Nb elements eliminates microstructural segregation. The formation of carbon–nitride compounds hinders the growth of austenite grains, refining and homogenizing the microstructure, which maximizes the strength. At 820–860 °C, the degree of austenitization increases with increasing annealing temperature, resulting in the formation of quenched martensite through rapid cooling. These martensites then temper during the subsequent over-aging holding stage, leading to a reduction in tensile strength [9,41]. The elongation of the test steel increases continuously with the rise in heating temperature. In the early stage, as the annealing temperature increases, the ferrite grains become finer and more uniform, and the banded microstructure gradually disappears, resulting in increased elongation of the test steel [42]. In the later stage, the tempering effect of martensite also contributes to increasing elongation. Additionally, the TRIP effect from the increased amount of retained austenite at higher annealing temperatures further enhances specimen elongation.

**Figure 9:**
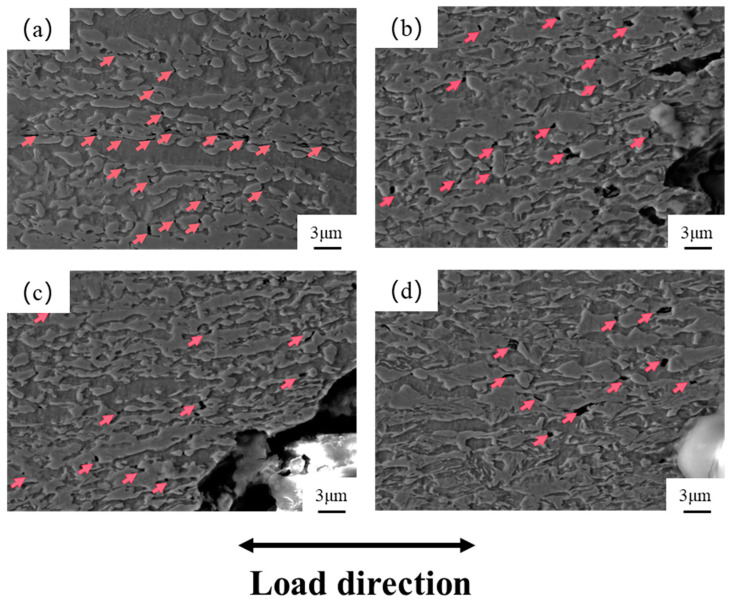
Micro-morphology of the vertical section of the tensile fracture: (**a**) 800 °C; (**b**) 820 °C; (**c**) 840 °C; (**d**) 860 °C.

In order to further analyze the fracture mechanism of the specimens under different annealing temperatures, micro-morphological observation was carried out on the vertical section of the tensile fracture, as shown in Figure 9. A large number of pores and secondary cracks can be observed near the fracture, as indicated by the red arrows. These are formed in areas with the highest stress concentration, such as between martensitic grains and between ferrite and martensite [43,44], and extend along the rolling direction. From the figure, it can be seen that the number of pores in the tensile specimens annealed at 800 °C is higher, and there is a tendency to form cracks along the rolling direction. In contrast, the number of pores in the specimens annealed at 820–860 °C gradually decreases. This is because, at higher annealing temperatures, the grains become finer and more uniform, and the deformation of the various phases is more coordinated [45,46]. As pointed out by Tang and Debdulal [47,48], during the deformation process, an uneven distribution of stress and strain occurs between grains. The soft phase deforms before the hard phase, and the strain is transferred to the hard phase. When the hard phase reaches its deformation limit, it will co-deform with the soft phase, eventually leading to the formation of micro-holes in the hard phase. The stress concentration at the grain boundary causes the microcrack to expand and cross into the next grain.

## 4. Conclusions

(1) The amount of tempered martensite decreased, but the degree of tempering increased with the rising overage temperature. Higher aging temperatures generated bainite structures and increased the amount of retained austenite. Consequently, the tensile and yield strengths of the test steels decreased, while the elongation increased. With the annealing temperature kept constant at 840 °C, the best strength–ductility balance was achieved at an aging temperature of 310 °C, resulting in a tensile strength of 1258 MPa and an elongation of 12.4%.
(2) As the annealing temperature increases, the banded structures disappear, and the grains become finer and more uniform. The number of precipitates is higher at elevated annealing temperatures. The yield and tensile strengths of the test steels initially increase and then decrease, while the elongation continuously increases. With the over-aging temperature kept constant at 310 °C, the best strength–ductility balance was achieved at an annealing temperature of 860 °C, resulting in a tensile strength of 1209 MPa and an elongation of 14.6%. At higher annealing temperatures, grain deformation is more harmonized, and the number of pores is reduced.

## Figures and Tables

**Table 1:** Chemical composition of tested steels (wt.%).

C	Mn	P	S	Si + Al	Cr	V	Nb
0.19–0.23	1.90–2.50	≤0.010	≤0.006	0.50–1.50	0.20–0.50	0.06–0.10	0.02–0.05

## Data Availability

The original contributions presented in the study are included in the article, further inquiries can be directed to the corresponding author.
